# Lessons learned from the microbial ecology resulting from different inoculation strategies for biogas production from waste products of the bioethanol/sugar industry

**DOI:** 10.1186/s13068-016-0548-4

**Published:** 2016-07-16

**Authors:** Athaydes Francisco Leite, Leandro Janke, Hauke Harms, Hans-Hermann Richnow, Marcell Nikolausz

**Affiliations:** Department of Environmental Microbiology, Helmholtz Centre for Environmental Research-UFZ, Permoserstrasse 15, 04318 Leipzig, Germany; Department of Biochemical Conversion, Deutsches Biomasseforschungszentrum gemeinnützige GmbH, Torgauerstrasse 116, 04347 Leipzig, Germany; Department of Isotope Biogeochemistry, Helmholtz Centre for Environmental Research-UFZ, Permoserstrasse 15, 04318 Leipzig, Germany

**Keywords:** Inoculation, Biogas process, Cattle manure, Bioethanol/sugar waste, 454 Pyrosequencing, Methanogens

## Abstract

**Background:**

During strategic planning of a biogas plant, the local availability of resources for start-up and operation should be taken into consideration for a cost-efficient process. Because most bioethanol/sugar industries in Brazil are located in remote areas, the use of fresh cattle manure from local farms could be a solution for the inoculation of the biogas process. This study investigated the diversity and dynamics of bacterial and archaeal communities and the performance of biogas reactors inoculated with manure and a mixed inoculum from different biogas reactors as for a controlled start-up until steady state.

**Results:**

Laboratory-scale biogas reactors were fed semi-continuously with sugarcane filter cake alone (mono-digestion) or together with bagasse (co-digestion). At the initial start-up, the reactors inoculated with the mixed inoculum displayed a less diverse taxonomic composition, but with higher presence of significant abundances compared to reactors inoculated with manure. However, in the final steady state, the communities of the differently inoculated reactors were very similarly characterized by predominance of the methanogenic genera *Methanosarcina* and *Methanobacterium*, the bacterial families *Bacteroidaceae*, *Prevotellaceae* and *Porphyromonadaceae* (phylum *Bacteroidetes*) and *Synergistaceae* (phylum *Synergistetes*). In the mono-digestion reactors, the methanogenic communities varied greater than in the co-digestion reactors independently of the inoculation strategy.

**Conclusion:**

The microbial communities involved in the biogas production from waste products of the Brazilian bioethanol/sugar industry were relatively similar and stable at the reactor’s steady phase independently of the inoculum source (manure or mixed inoculum). Therefore, the locally available manure can be used as inoculum for start-up of the biogas process, since it also contains the microbial resources needed. The strong fluctuation of methanogenic communities in mono-digestion reactors indicates higher risk of process instability than in co-digestion reactors.

**Electronic supplementary material:**

The online version of this article (doi:10.1186/s13068-016-0548-4) contains supplementary material, which is available to authorized users.

## Background

The Brazilian bioethanol/sugar industry has been previously reported in our studies to have a big potential to improve the local bioeconomy while reducing greenhouse gas emission by applying biogas technology to the treatment of its waste products [1–3]. However, to make the biogas technology profitable and reliable for the Brazilian bioethanol/sugar industry, the reactor design and start-up requires strategic considerations.

In regions where biogas plants are widespread and well developed, a biogas reactor can be started with inocula from already established processes. Contrary to this scenario, in Brazil there are only very few plants applying the anaerobic digestion (AD) process to treat waste at large scale and these plants are spread across the country and separated by long distances. Therefore, we have addressed the possibility of reactor inoculation with fresh cattle manure (FCM) as a locally available, potential inoculum. For comparison in terms of microbial robustness, we have also prepared an engineered mixed inoculum (referred to as MIX) originating from the digestate of different biogas reactors fed mainly with energy crops and agricultural wastes such as maize silage, thin stillage, straw and chicken manure. The first results regarding biogas production and feasibility of these two different inoculation strategies were recently reported by Janke et al. [4]. Nevertheless, the microbiological background of this experiment remained to be investigated.

In the biogas process, a complex metabolic network of microorganisms is responsible for organic matter degradation, which proceeds in four steps: hydrolysis, acidogenesis, acetogenesis and methanogenesis. While bacteria are involved in the first three steps, methanogenic archaea are responsible for the last step. Methanogens are very sensitive to process changes due to the relative lack of functional redundancy and low diversity [5]. Besides that, the methanogens are very important for the AD process stability, because they are directly involved in the removal of fermentation product acetate or makes the syntrophic oxidation of acetate and other fermentation products thermodynamically feasible by keeping hydrogen partial pressure low [6]. Therefore, the methanogenic community requires particular regard for the development of an efficient and robust AD process.

The methane formation by the methanogens is carried out either via direct acetate conversion (aceticlastic methanogenesis) or reduction of CO_2_ with H_2_ (hydrogenotrophic methanogenesis). The determination of different methanogenic pathways has been described to have crucial implications for the design and operation of biogas reactors [5, 7–10], since the aceticlastic and hydrogenotrophic methanogens may have different growth rates depending on the reactor’s conditions [11]. However, the factors controlling the balance of methanogenic pathways is still not clear and seems to depend on the substrate used and process conditions such as organic loading rate, reactor type and temperature [12–14].

Knowledge of microbial adaptation to environmental conditions is also very important to understand the complex interplay between bacteria and methanogens [15, 16], particularly during acclimatization leading to successful and efficient reactor operation [17]. Moreover, correlations of methanogenic community data with process parameters have been reported to contain decisive information about shifting pathway dominance during biogas production [9, 18, 19].

In a comprehensive study, we investigated the impact of different inoculation strategies and digestion setups (mono-digestion of filter cake and its co-digestion with bagasse) on the microbial community composition and dynamics along the operation of six mesophilic laboratory-scale continuously stirred tank reactors. In addition, the bacterial and methanogenic communities were assessed to identify key microorganisms of indicator value for the AD of the waste products from the bioethanol/sugar industry. Moreover, this study evaluated the links between microbial composition and reactor performance as a basis for an efficient future microbial resource management. The microbial communities were analyzed by DNA-based fingerprinting techniques and next-generation sequencing. The correlation between the microbial communities and reactor parameters was established by multivariate data analysis. In addition, the dominant methanogenic pathway was determined by the stable isotope fingerprinting of the produced biogas, following approaches applied to various anaerobic systems [20] and biogas reactors with several substrates [7, 21, 22].

## Methods

### Biogas reactors

**Fig. 1:**
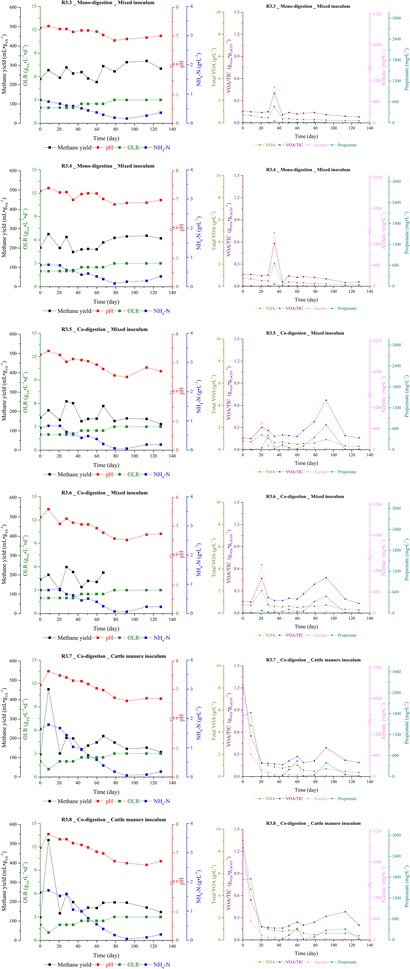
Major process parameters during the anaerobic digestion of filter cake and its co-digestion with bagasse in different inoculation strategies (MIX and FCM). The data showed in this figure was adapted from our previous study [4]. The process parameters data corresponds to the same sampling time of the digestate and the biogas for the molecular and isotopic analyses, respectively

Six identical laboratory-scale continuously stirred tank reactors with working volumes of 3 L under mesophilic conditions (38 ± 1 °C) were established to operate three experiments in duplicate: R3.3 and R3.4 were inoculated with MIX and fed with a single substrate (mono-digestion of filter cake); R3.5 and R3.6 obtained the same inoculum, but the filter cake was co-digested with bagasse; R3.7 and R3.8 were inoculated with FCM and performed co-digestion similarly to R3.5 and R.3.6. Figure 1 shows the major process parameters for each reactor along the whole experiment. The analytical methods and detailed description of the start-up and performance of the reactors are reported in our previous study [4]. Briefly, the experiments of 137 days duration were divided into two phases: start-up (until day 69) and steady state (from day 70 on), as shown in Table 1. In our experiments, the day 0 does not represent the inoculation with MIX and FCM, but the end of an acclimatization time of around 24 h. On day 0, the feeding of the reactors was started. During the start-up phase, the organic loading rate (OLR) varied from 1.0 (only R3.7 and R3.8) or 2.0 (the other four reactors) to 2.5 g_vs_ L^−1^ days^−1^ (all reactors), whereas during the steady state the OLR was constant at 3.0 g_vs_ L^−1^ days^−1^. The hydraulic retention times (HRT) at the final stage of the steady state were 23 and 28 days for mono- and co-digestion, respectively.

**Table 1:** Feeding regime divided into start-up and steady state according to variations on HRT and OLR. This table was adjusted from our previous study [4]

Reactors	Inoculum	C:N ratio	Phase	Period (day)	Filter cake	Bagasse	Water	HRT (days)	OLR (g_vs_ L^−1^ d^−1^)
					input (g day^−1^)	input (g day^−1^)	input (mL day^−1^)		
R3.3 R3.4	MIX	24:1	Start-up	Initial (0–41)	36.5	–	50	34.7	2.0
				Final (42–69)	45.7	–	50	31.4	2.5
			Steady	Initial (70–113)	54.8	–	50	28.6	3.0
				Final (114–137)	54.8	–	75	23.1	3.0
R3.5 R3.6	MIX	41:1	Start-up	Initial (0–41)	15.78	6.78	50	41.4	2.0
				Final (42–69)	19.73	8.45	50	38.4	2.5
			Steady	Initial (70–113)	23.67	10.15	50	35.8	3.0
				Final (114–137)	23.67	10.15	75	27.6	3.0
R3.7 R3.8	FCM	41:1	Start-up	Initial I (0–3)	15.78	6.78	50	41.4	2.0
				Initial II (4–20)	7.89	3.39	25	82.7	1.0
				Initial III (21–41)	15.78	6.78	50	41.4	2.0
				Final (42–69)	19.73	8.45	50	38.4	2.5
			Steady	Initial (70–113)	23.67	10.15	50	35.8	3.0
				final (114–137)	23.67	10.15	75	27.6	3.0

### Microbial community analysis

Duplicate digestate samples from each reactor were taken for molecular analysis on specific days and stored at −20 °C until further analysis. Simultaneously, biogas samples were taken from the reactors’ headspace for stable isotope analysis. The total genomic DNA of the bacterial and methanogenic communities was extracted with the ‘NucleoSpin Soil’ kit (Macherey–Nagel) as recommended by the supplier. The buffers SL2 and SX were used.

PCR amplifications for terminal restriction fragment length polymorphism (T-RFLP) screening of the methanogenic community were targeting the *mcrA* genes using the forward primer mlas and the reverse primer mcrA-rev and following the PCR protocol of Steinberg and Regan [23]. T-RFLP analysis of purified PCR products was conducted after digestion with the restriction enzyme *Bst*NI using the fragment size standard GeneScan-500 ROX (Applied Biosystems GmbH, Weiterstadt, Germany). T-RFLP electropherograms were processed as described by Lucas et al. [24]. During statistical analysis in R, signals with low peak areas were removed using a cutoff of 12 times the standard deviation of the data sets. The reproducibility of the T-RFLP was validated by comparing the results with duplicate samples from each reactor at a particular sampling day (Additional file 1: Figure S1). The *mcrA*-derived T-RFs were assigned taxonomically using cloned *mcrA* amplicons database from anaerobic digester sample analyses performed in our laboratory [21, 24–27].

The bacterial community analysis was performed only for the four co-digestion reactors inoculated with either FCM or MIX. Samples from two times (days 0 and 44) in the start-up phase and from one time (day 113) of the steady state were processed based on the 16S ribosomal RNA genes and further analyzed on the 454-pyrosequencing platform GS Junior (Roche) as described by Ziganshin et al. [28]. The variable regions V1–V3 of the bacterial 16S rRNA gene fragments were amplified with the primers Bac27F (5′-AGAGTTTGATCMTGGCTCAG-3′) and Bac519R (5′-GWATTACCGCGGCKGCTG-3′) using the Phire Hot Start II DNA Polymerase (Thermo Scientific). The raw sequence data were assessed with the QIIME 1.8.0 Virtual Box release [29]. Further data processing was performed according to Lucas et al. [24] and Sun et al. [14]. In summary, the dataset was firstly quality filtered by excluding sequences that were shorter than 150 and longer than 590 bp in lengths, comprised an average quality score below 25, held 50 bp at the end section below the quality score threshold of 25, comprised ambiguous bases, held a homopolymer run with more than 6 bp, or did not comprise any primer or barcode sequence. The USEARCH pipeline was applied on the sequences for further quality filtering based on non-chimeric sequences and for clustering into operational taxonomic units (OTUs) consisting of 97 % identity threshold [30]. The taxonomic classification based on representative sequences was performed using the Greengenes core set (gg_13_8) [31] and the Ribosomal Database Project classifier 2.2 [32]. For the taxonomic alignment, the Infernal algorithm with default setting was used [33]. Finally, the summarized OTU tables were constructed according to their taxonomy and abundance. Further, the visualization of the OTU tables was processed via the spreadsheet program. De-multiplexed sequences of the 12 samples were deposited under the EMBL-EBI accession number PRJEB12073 (http://www.ebi.ac.uk/ena/data/view/PRJEB12073). The ecological data analyses leading to chao1, Shannon and Simpson indices [34, 35] and rarefaction curve were also performed with the QIIME software based on alpha diversity. Due to the differences in sequencing library size between the samples, we have used QIIME further to subsample (rarefy) the libraries down to 8000 sequences per sample for comparative diversity analyses and to calculate the beta diversity (pairwise sample dissimilarity).

The ordination of the dissimilarity matrices achieved by non-metric multidimensional scaling (NMDS) was processed as reported by Lucas et al. [24]. Shortly, the variability of the microbial communities was evaluated by the Bray–Curtis dissimilarity index based on the presence and relative abundance. Thus, e.g., highly similar community composition is indicated by tiny distances. The correlation of reactor parameters and microbial communities based on the relative abundance was analyzed with the ‘envfit’ function and its significance was tested by a Monte Carlo test with 999 permutations. The significance threshold was set to a maxima of 0.001 and 0.05 for the methanogenic and the bacterial communities, respectively.

### Stable isotope fingerprinting

The carbon and hydrogen stable isotope compositions of CH_4_ and CO_2_ from each reactor were measured in triplicate biogas samples collected in 20-mL gas-tight pre-evacuated vials. For analysis, an isotope ratio mass spectrometry system (Finnigan MAT 253, Thermofinnigan Bremen) coupled to a gas chromatograph (GC) (HP 6890 Series, Agilent Technology, USA) via a combustion device and a pyrolysis unit (with a water-removal assembly) was used for carbon and hydrogen measurements, respectively. Fifty-microliter biogas sample was injected into the inlet tube of the GC instrument equipped with a CP-Porabond Q column (50 m × 0.32 mm ID Varian, USA) held at a constant temperature of 40 °C. Helium was used as a carrying gas at the split ratio of 1:50 for carbon and 1:5 for hydrogen analysis. The isotope ratios of all samples are given in delta notation (δ^13^C and δ^2^H) in per mil (‰) units according to the standards VPDB (Vienna Pee Dee Belemnite) for carbon and VSMOW (Vienna Standard Mean Ocean Water) for hydrogen.

## Results and discussion

### Bacterial community succession

**Fig. 2:**
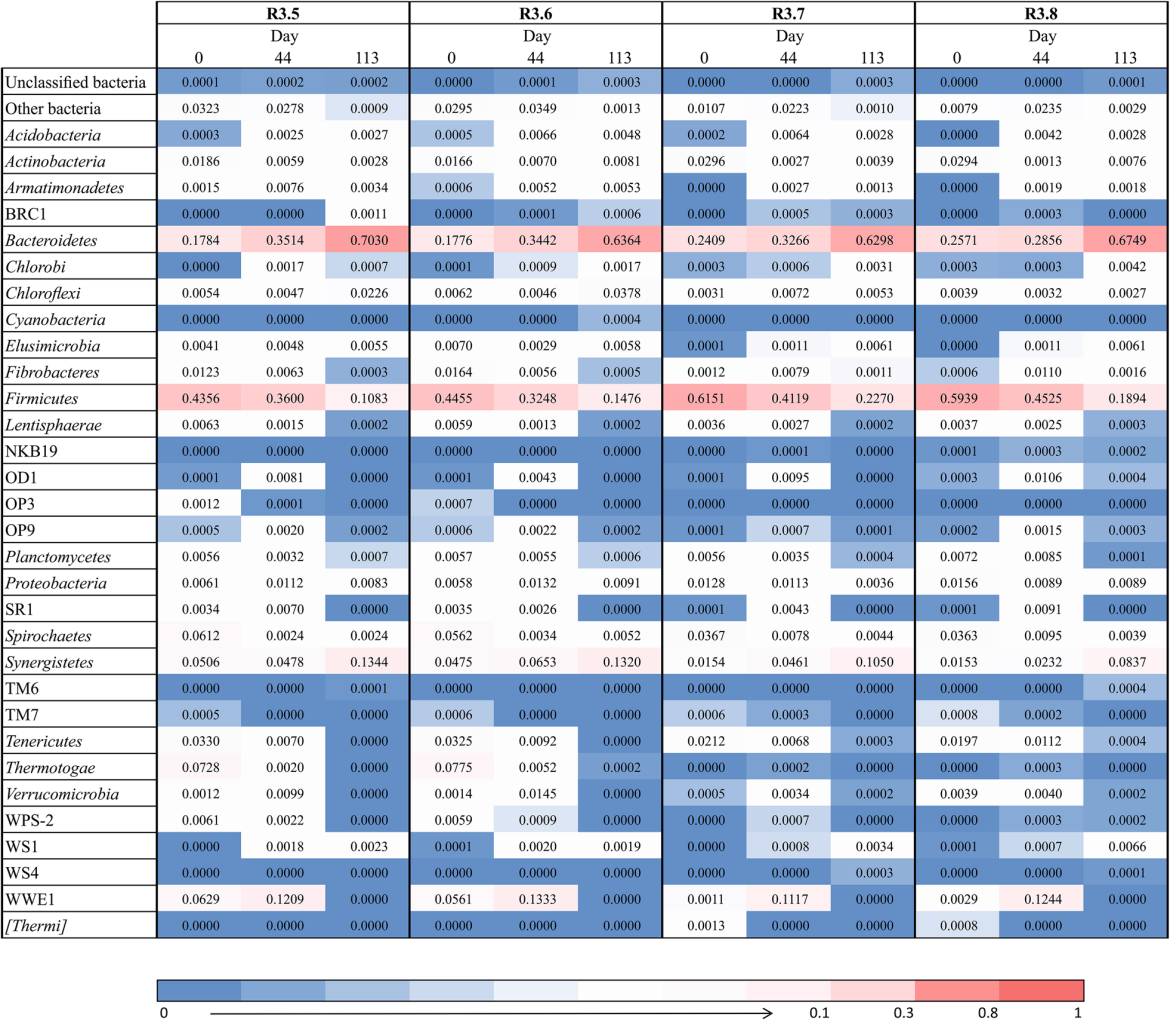
Phylogenetic composition and succession of the bacterial communities. Heatmap displaying the relative abundance of the bacterial communities at the phylum level along the experiment using different inoculation strategies (MIX and FCM) for the co-digestion of filter cake and bagasse. The relative abundances were based on the 454-pyrosequencing of 16S ribosomal RNA gene amplicons

The microbial profiles of anaerobic digesters have been reported to be very specific for each type of reactor and substrate feeding [12, 28, 36]. Thus, to investigate the shaping forces of novel substrates (filter cake and bagasse) on the inocula (FCM and MIX), the bacterial community in the co-digestion reactors (R3.5, R3.6, R3.7 and R3.8) was assessed by amplicon pyrosequencing at three sampling times, i.e., days 0 and 44 in the start-up phase and day 113 during steady state. The bacterial community succession of each reactor is shown on phylum level in Fig. 2. The parallel reactors R3.5 and R3.6, and R3.7 and R3.8 had very similar bacterial profiles. The community similarity between the reactor samples based on the beta diversity are shown in Additional file 1: Table S1 and Figure S3. All OTUs obtained from the 12 samples are presented in Additional file 2: Table S2, and the respective rarefaction curves are shown in the Additional file 1: Figure S2.

**Fig. 3:**
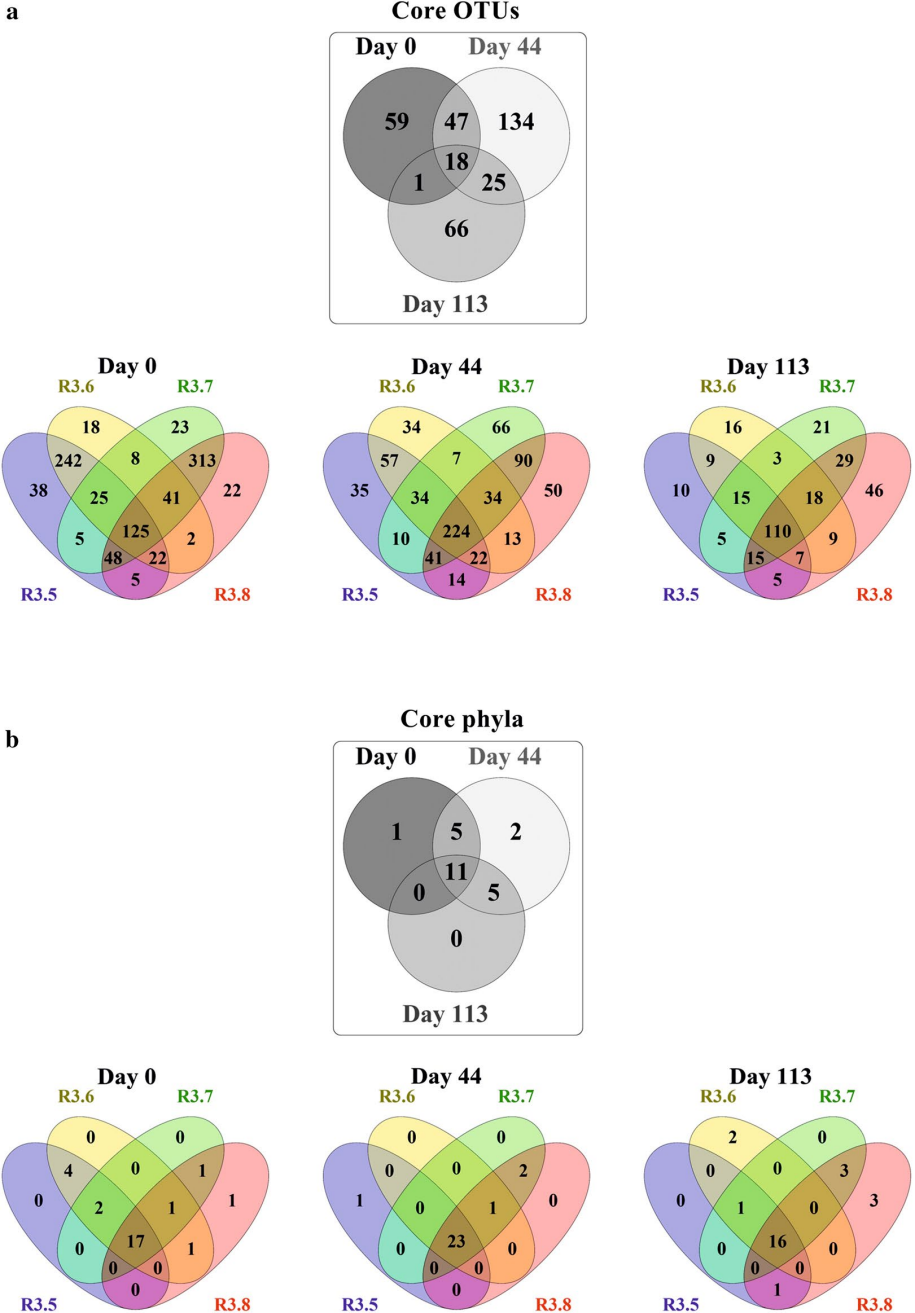
Venn diagram of the core OTUs (**a**) and phyla (**b**) of the bacterial communities. Three distinct sampling times during experiments with different inoculation strategies (MIX and FCM) for the co-digestion of filter cake and bagasse were assessed. The 18 core OTUs in the *greyscale* Venn diagram were from the 125, 224 and 110 core OTUs identified in the four reactors at day 0, 44 and 113, respectively. In the same way, the 11 core phyla presented also in a *greyscale* Venn diagram were from the 17, 23 and 16 core phyla found in all reactors at day 0, 44 and 113, respectively. The Venn diagrams were prepared according to Oliveros [52]

We obtained on average around 12,000 high-quality sequence reads per reactor, varying from circa 9000 to 13,000. Along the operation of the co-digestion reactors during start-up and steady state, the taxonomic composition of our analyzed reactors was very diverse with a total of 1137 OTUs, but only 18 core OTUs (1.6 %), which were found in all reactors at all sampling times (Fig. 3), accounting for 18 % of all sequence reads. Thus, the variation of bacterial communities was very high with only a few microorganisms being significantly abundant in all reactors throughout the experiment. Examples were the families *Porphyromonadaceae* and *Synergistaceae* accounting for 8 and 4 % of all sequence reads, respectively.

Figure 3 also shows the variation of the numbers of core OTUs and phyla along the experiment. Day 0 samples contained the highest numbers of unique OTUs shared either only by the MIX parallels R3.5 and R3.6 (242, 21 % of all OTUs) or by the FCM parallels R3.7 and R3.8 (313, 28 % of all OTUs). The parallel FCM reactors R3.7 and R3.8 were more diverse in terms of OTUs, but only few microorganisms of high relative abundance were detected. On the other hand, parallel MIX reactors R3.5 and R3.6 presented lower diversity comprising microorganisms with high relative abundances (Fig. 2). On day 44, the number of total shared OTUs and phyla for all reactors was significantly increased, whereas the amount of unique OTUs per parallel reactors was drastically decreased (57 for R3.5 and R3.6 and 90 for R3.7 and R3.8). On day 113, the number of total OTUs and phyla shared by all reactors was decreased to 110 and 16, respectively. Moreover, the number of unique OTUs shared by parallel reactors was much lower.

These variations indicate that despite different inoculation, the bacterial communities were already very similar after 44 days of reactor operation. Nevertheless, the consolidation and stabilization of the bacterial communities proceeded until the steady state. Figure 3 shows that there was uniqueness in terms of OTUs and phyla of individual reactors along the experiment, although R3.5 and R3.6 as well as R3.7 and R3.8 were operated in parallel under the same conditions.

The estimated richness of the bacterial community in all samples is shown in Table 2. The number of OTUs decreased slightly during start-up, whereas at steady state a drastic drop was observed. The same trend was reflected by the OTU richness estimator chao 1 and the Shannon index. The Simpson diversity was stable between the two samplings during start-up, while it decreased during steady state. In MIX reactors R3.5 and R3.6, the Simpson index dropped more than in the FCM reactors R3.7 and R3.8, indicating higher community evenness in the former. Generally, the bacterial species richness was lower during steady state with more pronounced predominance of some microorganisms.

**Table 2:** Ecological index showing the estimated richness of the bacterial communities

Reactor	Day	OTUs		Chao1		Shannon		Simpson	
R3.5	0	510	(441)	590	(562)	6.35	(6.35)	0.96	(0.96)
R3.6		483	(433)	548	(545)	6.33	(6.34)	0.96	(0.96)
R3.7		588	(554)	635	(621)	6.63	(6.64)	0.97	(0.97)
R3.8		578	(545)	607	(620)	6.69	(6.71)	0.97	(0.97)
R3.5	44	437	(400)	528	(521)	6.03	(6.03)	0.96	(0.96)
R3.6		425	(412)	538	(519)	6.24	(6.24)	0.96	(0.96)
R3.7		506	(462)	632	(597)	6.52	(6.48)	0.97	(0.97)
R3.8		488	(414)	595	(502)	6.36	(6.34)	0.97	(0.97)
R3.5	113	176	(156)	200	(194)	3.80	(3.76)	0.82	(0.82)
R3.6		187	(169)	227	(209)	4.06	(4.05)	0.86	(0.86)
R3.7		216	(181)	258	(222)	4.33	(4.32)	0.90	(0.90)
R3.8		239	(226)	316	(283)	4.58	(4.58)	0.90	(0.90)

Values in parentheses originated from all randomly subsampled (without replacement) libraries down to the lowest number of sequences per sample

The phyla *Firmicutes* and *Bacteroidetes* dominated the bacterial communities in all analyzed reactors along the entire operation time (Fig. 2). Both together comprised 72 % of all sequence reads. The presence of most OTUs affiliated with these two phyla known to utilize carbohydrates has frequently been reported in studies about AD of different substrates, mainly maize silage and manure, in laboratory- and full-scale biogas reactors [12, 24, 28, 36, 37].

Although the succession toward steady state was not favorable for *Firmicutes*, the phylum was the most diverse taxonomic group in the reactors with 620 OTUs (54 %), represented mainly by the order *Clostridiales* (485 OTUs), in which the families *Ruminococcaceae* (153 OTUs), *Clostridiaceae* (53 OTUs) and *Lachnospiraceae* (41 OTUs) were predominant. Following the succession of these families during our experiment, *Ruminococcaceae* was the most constant family, without much variation in its abundance (around 5 % in all reactor samples). *Clostridiaceae* had also a constant abundance around 0.8 % in the reactors inoculated with MIX. However, the same family was the most variable one in the reactors inoculated with FCM, presenting 21.8 and 1.3 % relative abundances at the beginning (day 0) and end of the experiment (day 113), respectively. The family *Lachnospiraceae* was relatively constant at abundances around 1.8 %, except for the reactors at the beginning of the experiment with MIX. Hence, our results with sugarcane waste products were in agreement with former studies that also investigated the bacterial communities in the AD of plant-based biomass [37, 38], especially in terms of the prevalence of *Clostridiales*. Members of this order are equipped with the cellulosome, a multienzyme complex, which enables them to efficiently hydrolyze recalcitrant cellulosic and hemicellulosic structures in the plant cell wall [39]. Our feeding substrates from the sugarcane industry contain high percentages of cellulose and hemicellulose, both together representing 75 and 55 % of the total carbohydrate and lignin content of bagasse and filter cake, respectively [1]. Sequences affiliated to the cellulosome-producing bacterium *Ruminococcus flavefaciens* were detected in our reactors. These type of sequences related to the anaerobic cellulolytic rumen bacterium contributed significant proportions of sequence reads in our experiment (0.79 % of all), especially at the end at day 113 (0.34 %), which suggests that this phylotype was one of the specialists degrading bagasse and filter cake, since both inocula sustained its presence. Members of the genus *Ruminococcus* are also known to produce hydrogen and acetate, thus supplying the hydrogenotrophic methanogenic genus *Methanobacterium* [40].

*Bacteroidetes* as the second most diverse phylum with 128 OTUs showed a gradual increase in relative abundance toward steady state. Within this phylum, OTUs affiliated with the genera *Bacteroides* (4 OTUs, ca. 9.4 % of all sequence reads) and *Prevotella* (4 OTUs, ca. 7.2 % of all reads) and the family *Porphyromonadaceae* (39 OTUs, ca. 9.8 % of all reads) were predominant. Since most of these sequence reads were detected at the end of the experiment, we assume that these microorganisms are crucial for the hydrolysis and fermentation of the filter cake and bagasse. The genus *Bacteroides* represented by the species *B. cellulosolvens* [41] is also known for its cellulosome. Within the genus *Prevotella*, there are some species notably involved in the degradation of hemicellulose, e.g., *Prevotella paludivivens* [42].

On day 0, the phyla *Firmicutes* and *Bacteroidetes* together represented about 85 and 62 % abundance in the reactors inoculated with FCM and MIX, respectively (Fig. 2). In R3.5 and R3.6 (MIX), other phyla such as *Actinobacteria*, *Fibrobacteres*, *Spirochaetes*, *Synergistetes*, *Tenericutes*, *Thermotogae* and the candidate WWE1 were also presented in significant relative abundances. Most of these phyla are involved in the degradation of lignocellulose-rich substrates [14, 28, 36]. However, the ecophysiological role of some phyla such as WWE1 is still unclear [24].

On day 44, the bacterial communities were already very similar in all reactors independent of the inoculation source. The relative abundance of the phylum *Firmicutes* decreased, whereas that of the *Bacteroidetes* increased. While the relative abundance of *Synergistete*s was kept constant in R3.5 and R3.6 (MIX), it increased slightly in the other co-digestion reactors (R3.7 and R3.8). The third most abundant phylum at this time was the candidate WWE1 (12 %), which had been found in many other studies dealing with mesophilic AD in the frame of wastewater treatment [43, 44]. Moreover, this candidate phylum was recently reported in large-scale continuously stirred tank reactors digesting the cellulose-rich substrate maize silage [24].

The steady state on day 113 was characterized by a less diverse community with three major phyla, *Bacteroidetes*, *Firmicutes* and *Synergistete*s, comprising nearly 94 % of the entire community. The relative abundance of the phylum *Bacteroidetes* of approximately 66 % in all reactors was even greater than on day 44. On the contrary, the abundance of *Firmicutes* dropped further to about 16 %. *Synergistete*s were present in all reactors along the experiment and their increasing abundance (to ca. 11 %) demonstrated their participation in the degradation of the lignocellulosic bagasse and filter cake. Within this phylum, the class S*ynergistia* has reported to degrade fiber-rich feedstock [36]. Whereas all reactors had a very similar overall community structure, the phylum *Chloroflexi* (specifically, class *Anaerolineae*) was significantly abundant (3 %) only in R3.5 and R3.6 (MIX). This phylum had also been found in similar proportion in a biogas plant co-digesting maize silage, green rye and liquid manure [37].

**Fig. 4:**
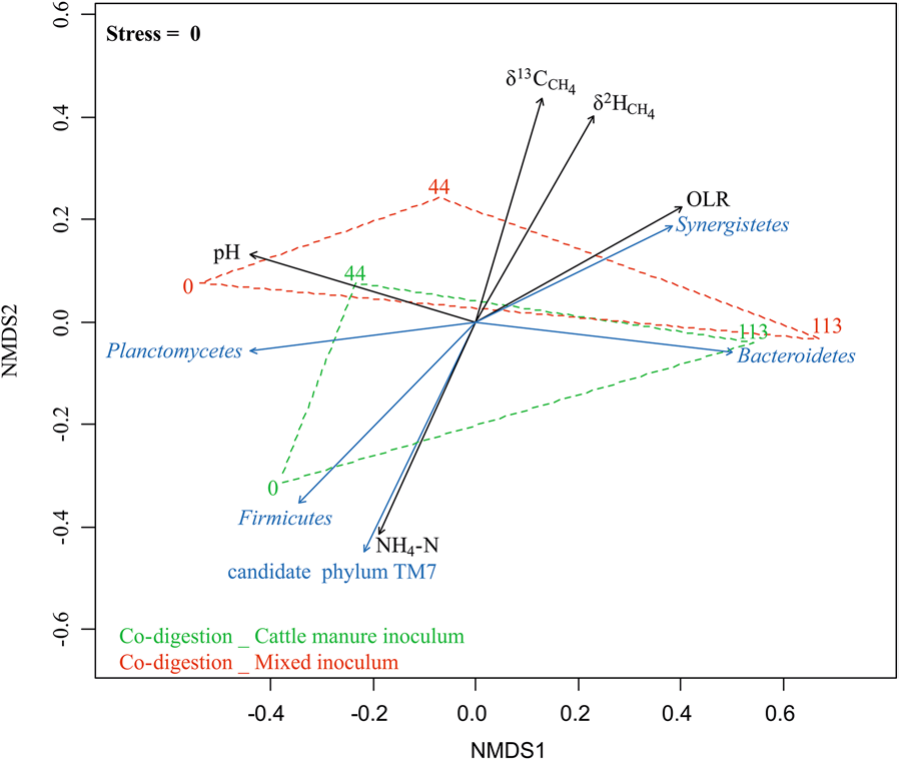
The NMDS plot analyses of the bacterial communities (phylum level). The results were based on the pyrosequencing data of the 16S ribosomal RNA genes. The data points are numbered according to specific sampling days. The *blue arrows* indicate the highly significant (*p* < 0.05) bacterial phyla, whereas the *black arrows* represent the highly significant (*p* < 0.05) reactor parameters as correlation vectors of the bacterial community succession. The arrow length shows the correlation with the ordination axis, while the arrow direction corresponds to the community structures

The plot in Fig. 4 shows an NMDS analysis of bacterial 16S rRNA genes. The bacterial community compositions notably converged toward steady state (day 113). It is also visible that duplicate FCM reactors presented more diverse bacterial communities than MIX reactors. The correlation between the bacterial communities and the reactor parameters is indicated by the most significant vectors represented as arrows in the NMDS plot. The abundance of the phylum *Synergistetes* was strongly correlated with increasing OLR, whereas the abundance of the candidate phylum TM7 grew with the decrease of NH_4_–N. The more enriched values for δ^13^C and δ^2^H of methane were inversely correlated with NH_4_–N and positively with the bacterial community presented after the second sampling point (day 44). Furthermore, the decrease of pH influenced the bacterial communities primarily in the start-up phase. As already mentioned, the presence of phylum *Bacteroidetes* was strongly correlated with the steady state (at day 113).

### Dynamics of the methanogenic community

**Fig. 5:**
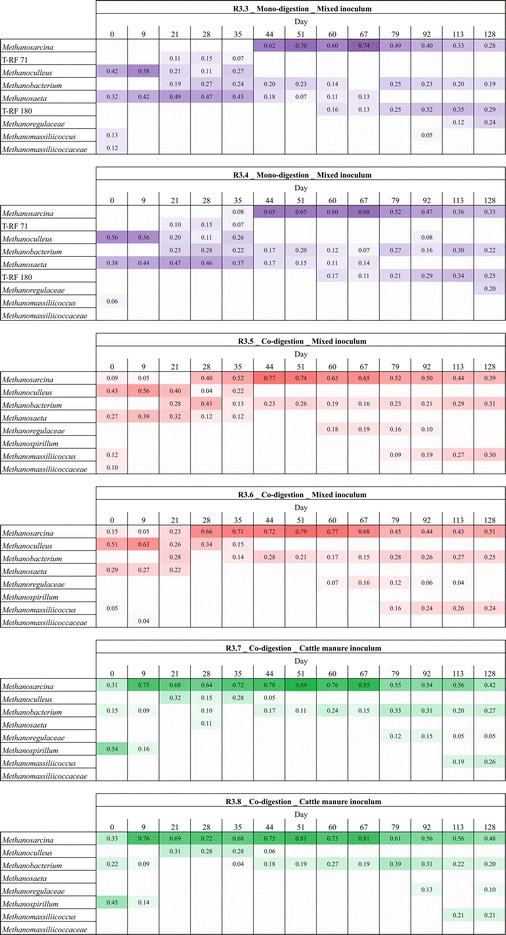
Heatmap composing the T-RFLP profiles of the methanogenic community dynamics. The relative abundances are shown according to the sampling days of the experiments with different inoculation strategies (MIX and FCM) and digestion setups with filter cake and bagasse. Color intensity increases with relative abundance. Two digestate samples for each reactor were analyzed on every sampling day. Due to the high similarity of these samples, an average was calculated to show a more representative T-RFLP profile of each reactor

The phylogenetic composition and dynamics of the methanogenic community were analyzed in all six reactors by *mcrA*-based T-RFLP fingerprinting. A heatmap presenting the relative abundance of the methanogenic communities along the whole experiment is shown in Fig. 5. Duplicate reactors (R3.3 and R3.4, R3.5 and R3.6, R3.7 and R3.8) had very similar methanogenic community compositions at each sampling time (Additional file 1: Figure S4). However, some non-abundant microorganisms, for examples *Methanomassiliicoccaceae* at the initial phase and *Methanomassiliicoccus* and *Methanoregulaceae* at the steady phase were occasionally detected in one of the duplicate reactors. Except during start-up, microorganisms affiliated with the genus *Methanosarcina* were the most abundant methanogens across all analyzed samples, reaching as much as 90 % T-RF abundance at day 51 in the final stage of the start-up phase.

The first sampling on day 0 was characterized by different methanogens in the MIX and FCM inocula. MIX-inoculated reactors were dominated by the genera *Methanoculleus* (ca. 50 %) and *Methanosaeta* (ca. 30 %) and less by *Methanomassiliicoccus* (ca. 8 %). *Methanosarcina* and *Methanomassiliicoccaceae* were not detected simultaneously in all four reactors at day 0. In the FCM-inoculated reactors, the most abundant genera were *Methanospirillum* (ca. 50 %), *Methanosarcina* (ca. 30 %) and *Methanobacterium* (ca. 20 %). In case of both inoculation strategies, the predominant methanogens were affiliated to either strictly hydrogenotrophic taxa or to the metabolically flexible *Methanosarcina* genus. Nevertheless, strict aceticlastic methanogens were also present at usually minor abundances.

The addition of bagasse as a co-substrate in MIX-inoculated reactors had an influence on the methanogenic community. The T-RF profiles of the mono- and co-digestion on day 28 were already significantly distinct. On the other hand, the methanogenic community structures of the co-digestion reactors (MIX and FCM) were similar after sampling day 21. In this latter case, the dynamics of the microbial community also followed the same trend toward the predominance of the *Methanosarcina*.

The genus *Methanosarcina* has been described to out-compete *Methanosaeta* at elevated acetate levels, due to its faster growth kinetics [45, 46]. However, recently *Methanosaeta* was reported to dominate over *Methanosarcina* even at high acetate concentration in the AD of dairy waste with poultry waste as co-substrate [47]. In accordance with this recent finding, the high abundance of the strictly aceticlastic *Methanosaeta* was stable during volatile organic acids (VOA) accumulation (1273 mg L^−1^ of acetate and 55 mg L^−1^ of propionate) at day 35. This corroborates our previous study [26], in which *Methanosaeta* was also detected at high VOA concentrations.

In the final part of the start-up phase (days 42–69), *Methanosarcina* was the most abundant methanogen reaching the highest T-RF relative abundance in all reactors. The presence of *Methanosarcina* in AD is regarded to be advantageous. As discussed by De Vrieze et al. [48], it is very robust against many stress conditions, e.g., tolerate process overloading, sudden pH changes and high levels of ammonium and salt. In addition, *Methanosarcina* has high growth rates, can use both the aceticlastic and the hydrogenotrophic pathway and utilize methanol to produce methane.

In the late start-up stage, *Methanoculleus* and *Methanospirillum*, which had been dominant in the MIX and FCM inocula, respectively, were no longer abundant. The taxon represented by T-RF 180 in the mono-digestion (MIX) and *Methanoregulaceae* in the co-digestion (MIX) became abundant until steady state.

The relative abundance of *Methanosarcina* decreased at steady state to around 30 and 50 % in the mono- and co-digestion reactors, respectively. In the mono-digestion, the taxon represented by T-RF 180 and *Methanobacterium* became more abundant, while *Methanoregulaceae* appeared first in the process. In the four co-digestion reactors, the methanogenic communities were very similar during steady state. Generally, the abundances of *Methanomassiliicoccus* and *Methanobacterium* increased, whereas *Methanoregulaceae* was not abundant.

**Fig. 6:**
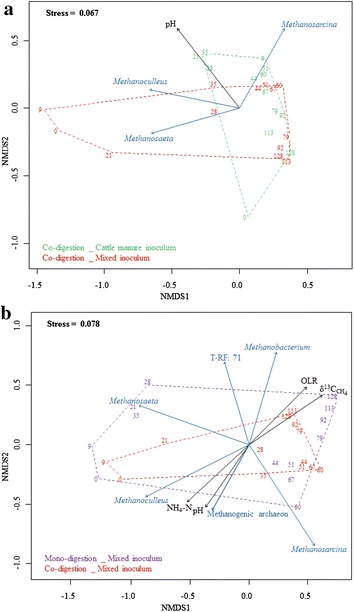
NMDS plot analyses of the methanogenic T-RFLP profiles and its correlation with reactor parameters. The hulls show the effect of the inoculation strategy (MIX vs. FCM) using the co-digestion setup (**a**), and the effect of the substrate (mono vs. co-digestion) applying MIX inoculum (**b**). The data points are numbered according to specific sampling days. The *blue arrows* indicate the highly significant (*p* < 0.001) methanogens, whereas the *black arrows* represent the highly significant (*p* < 0.001) reactor parameters as correlation vectors of the methanogenic community dynamics. The *arrow length* shows the correlation with the ordination axis, while the *arrow direction* corresponds to the community structures

Figure 6 shows the results of a multivariate statistical analysis based on the correlation between T-RLFP data and reactor parameters such as biogas yield and composition (CH_4_ and CO_2_), volatile fatty acids (acetate, propionate and butyrate), pH, total VOA, volatile organic acids per total inorganic carbonate buffer (VOA/TIC), HRT, ammonium–nitrogen (NH_4_–N) and stable isotope data (δ^13^C_CH4_, δ^13^C_CO2_ and δ^2^H_CH4_). The effect of the inoculation strategy (MIX vs. FCM) using the co-digestion setup and the effect of the substrate (mono vs. co-digestion) applying MIX inoculum were analyzed separately for more comprehensible results.

Figure 6a compares the different inoculation strategies (MIX and FCM) in the co-digestion setup. The main difference between them was observed at the beginning of the start-up phase. After day 21, both setups, independent of the inoculum, had attained similar methanogenic community patterns with minor temporal compositional shifts. *Methanoculleus* and *Methanosaeta* were strongly correlated with the MIX inoculum at the beginning of the experiment, whereas *Methanosarcina* was predominantly present in the second part of the start-up phase. The methanogenic community dynamics was most significantly correlated (*p* < 0.001) with the pH, which gradually decreased during the start-up phase and kept constant values during steady state. On day 128, the T-RFLP profiles clustered together, indicating very high similarity of the methanogenic community structures, independent of the inoculum source.

Mono- and co-digestion with the same inoculum (MIX) were compared in an independent analysis (Fig. 6b). Although the substrate regimes differed, methanogenic community patterns were similar. Bigger changes in the structure of the methanogenic community were observed during mono-digestion, visible as a more extended dashed hull, while the samples from the co-digestion reactor clustered more closely together. The reactor parameters with most significant correlation (*p* < 0.001) to the community structures of the investigated samples were OLR, δ^13^C_CH4_, NH_4_–N and pH. Whereas OLR and δ^13^C_CH4_ values rose along the reactor operation, NH_4_–N concentration and pH value decreased gradually, mainly in the start-up phase.

### Stable isotope characterization of the produced biogas

**Fig. 7:**
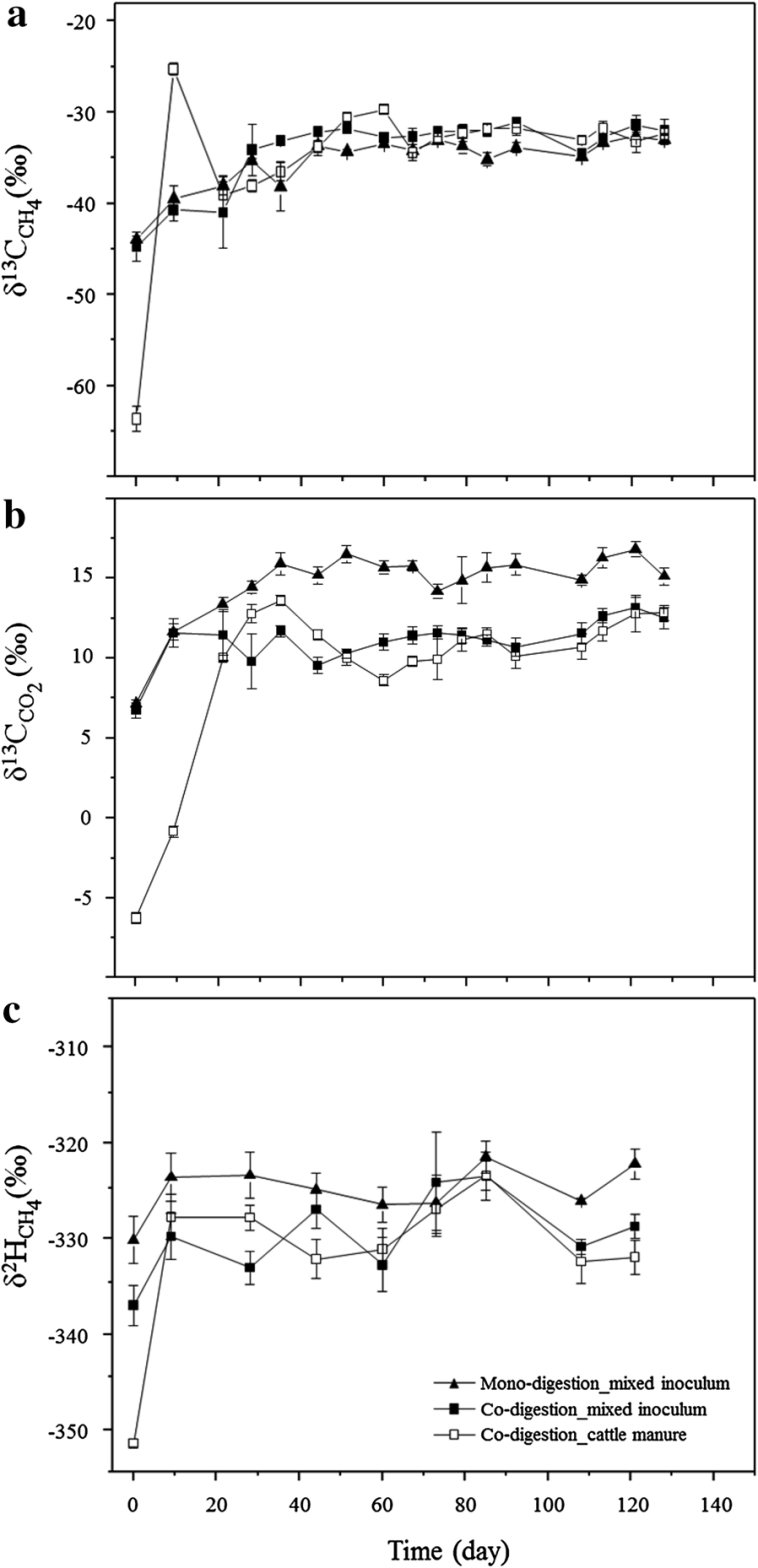
Biogas isotope composition dynamics. The isotope signatures were plotted according to δ^13^C (**a**) and δ^2^H (**c**) of methane and δ^13^C of carbon dioxide (**b**) along the experiment with different inoculation strategies and digestion setups. The *error bars* with the standard errors are shown with the confidence interval of 95 % for the δ^13^C values and 65 % for the δ^2^H values

Isotopic variations as a function of inoculation strategies (MIX and FCM) and substrate regimes are shown in Fig. 7. Samples for δ^13^C_CH4_, δ^13^C_CO2_ and δ^2^H_CH4_ measurements were taken together with samples for the analyses of microbial profiles and reactor parameters. Substantial isotopic changes were mainly observed in the initial start-up phase (days 0–41). Thereafter, the isotope values followed very similar trends independent of the inoculum. Initially, the isotope values were strongly depleted, particularly in reactors inoculated with FCM (−64 ‰ for δ^13^C_CH4_, −6 ‰ for δ^13^C_CO2_ and −352 ‰ for δ^2^H_CH4_).

Figure 7a shows that the δ^13^C_CH4_ values during the initial start-up increased until day 44. As an exception on day 9 in the FCM-inoculated reactors, the δ^13^C_CH4_ values changed drastically to −25 ‰, but from day 21 onward the δ^13^C_CH4_ values followed the same trend as in the other reactors inoculated with MIX. A similar isotope signature for δ^13^C_CH4_ of about −32 ‰ on day 44 had also been observed in our previous study of co-digestion of filter cake and bagasse at gradual OLR increase [26] and in some other studies with C4 plant biomass, namely maize silage [7, 21]. Afterward, all reactors displayed constant isotope signatures also indicating stable methanogenic process.

The effect of different inocula and substrates on the δ^13^C_CO2_ composition is clearly observed in Fig. 7b. The reactors inoculated with MIX had very similar isotope values on days 0 and 9. But after that, the isotopic pattern diverged between mono- and co-digestion. Enriched δ^13^C_CO2_ values were found for mono-digestion (up to about 15 ‰). The reactors inoculated with FCM had much more depleted δ^13^C_CO2_ values on days 0 and 9. On day 79 during steady state, the δ^13^C_CO2_ values for all co-digestion reactors were very similar (around 11 ‰).

The δ^2^H_CH4_ values shown in Fig. 7c presented a similar tendency among the reactors at specific sampling times. After start feeding the freshly inoculated reactors (from day 9 on), the δ^2^H_CH4_ values indicated enrichment of methane in deuterium. Similarly to the dynamics of δ^13^C_CO2_ values, the influence of the co-digestion was also visible from the variation of the δ^2^H_CH4_ isotopic signatures, mainly at the last samplings. In co-digestion reactors, lower δ^2^H_CH4_ values were observed.

The δ^13^C composition of filter cake and bagasse were −14.30 and −13.64 ‰, respectively [26]. The isotopic signature of the substrates has also influenced the trends observed in Fig. 7, in which the δ^13^C values became more enriched. The daily added tap water affects as well the δ^2^H_CH4_ isotopic signatures. Nikolausz et al. [7] reported that the δ^2^H of the tap water in Leipzig corresponds to about −64.7 ± 0.8 ‰. Therefore, more enriched δ^2^H_CH4_ values were found after the addition of water to the substrates.

### Methanogenic pathways

**Fig. 8:**
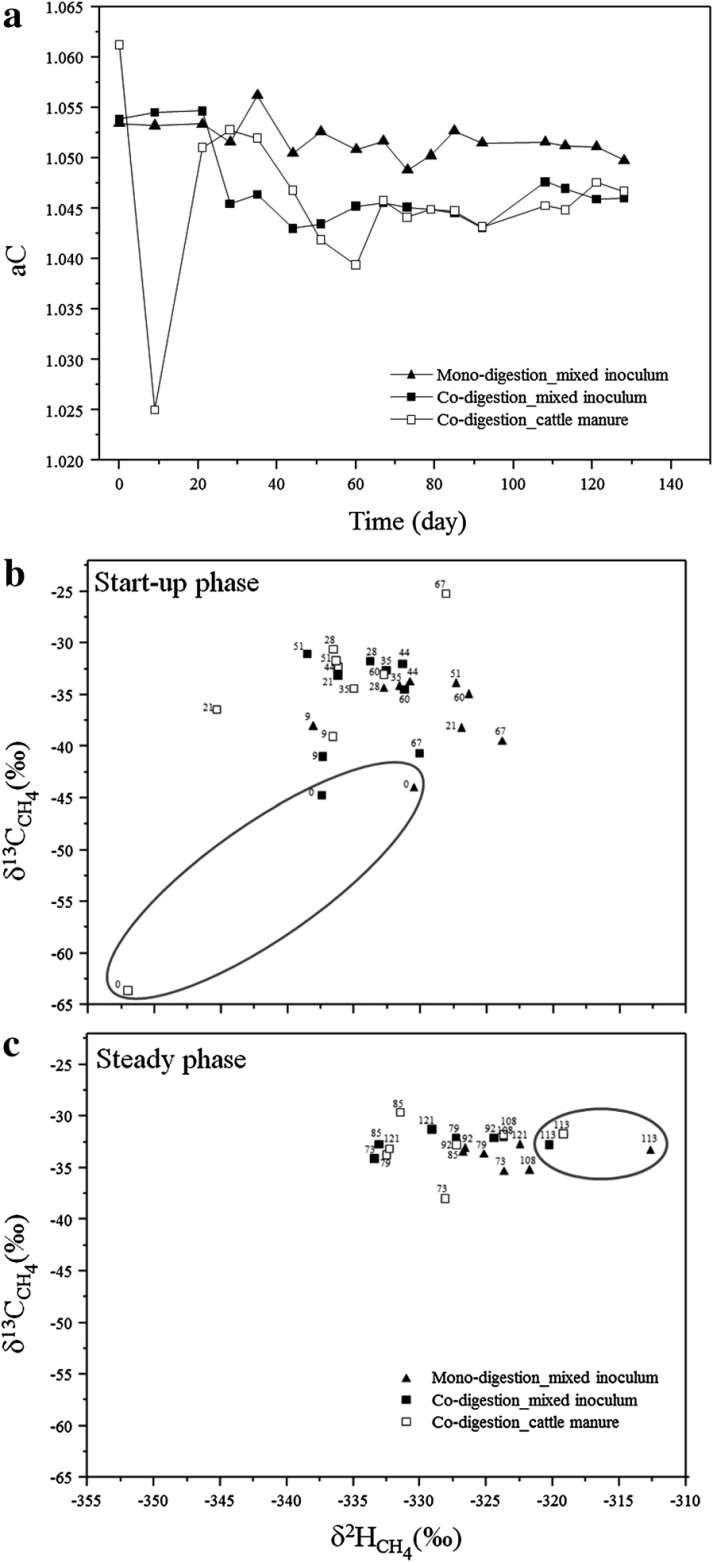
Determination of the potential predominant methanogenic pathway. The estimate dynamics shifts were based on the αC calculation (**a**) and on the correlation of δ^2^H and δ^13^C of methane. The *hulls* indicate the effect of the different inoculation strategies (**b**) and digestion setups (**c**) on the methanogenic pathways. The sampling days are plotted together with the representative shape point

The dominance of a specific methanogenic pathway along the reactor operation was assessed using the apparent fractionation factor (αC) (Fig. 8a), which was calculated based on the isotopic composition (δ^13^C) of methane and carbon dioxide. As previously reported [20, 49, 50], a predominance of hydrogenotrophic methanogenesis is indicated by αC >1.065, whereas αC <1.025 indicates the predominance of aceticlastic methanogenesis. Intermediate αC values mostly found along the experiment showed that both methanogenic pathways were involved in methane production. This is also in agreement with the methanogenic community profiles composed of hydrogenotrophic and aceticlastic methanogens (Fig. 5).

Only on days 0 and 9 in co-digestion reactors inoculated with FCM, the predominance of one methanogenic pathway was observed. On day 0, the αC value indicated the predominance of hydrogenotrophic methanogenesis in accordance with a 68 % predominance of the strictly hydrogenotrophic genera *Methanospirillum* (ca. 50 %) and *Methanobacterium* (18 %). In addition, the versatile genus *Methanosarcina*, which contributed 32 % to the community profile, most probably produced methane via the hydrogenotrophic pathway during this period. On day 9, the methanogenic pathway was shifted drastically to the dominance of aceticlastic methanogenesis. The αC value was slightly lower than 1.025 and the predominance of the versatile *Methanosarcina* (ca. 75 %) suggested that members of this genus became not just numerically abundant, but switched to methane production from acetate. On day 21, again a sudden change was observed from the αC dynamics. At this time, *Methanosarcina* was still the most abundant taxon, which, however, probably used both methanogenic pathways.

The methanogenic pathway dynamics during the experiment is possibly also observed in plots combining δ^13^C and δ^2^H isotopic variations of methane during start-up (Fig. 8b) and steady state (Fig. 8c). Nikolausz et al. [7] had used this combination of δ^13^C_CH4_ and δ^2^H_CH4_ to identify the dominant methanogenic pathway in laboratory-scale biogas reactors. Accordingly, the suggested predominance of the hydrogenotrophic methanogenesis at day 0 (marked in the graphic with the hull) for FCM-inoculated reactors is confirmed as suggested by Sugimoto and Wada [51]. Although their classification did not correspond to the other sampling points in terms of predominant methanogenic pathway defined by αC and the molecular biological results, the differences between the inocula and the substrate were still observed in the combined δ^13^C_CH4_ and δ^2^H_CH4_ graphics (hulls). A general trend of less negative δ^2^H_CH4_ values observed in case of later samples in all reactors is probably due to the influence of the isotopic composition of the supplemented process water.

## Conclusion

Our results confirmed that FCM is a reliable and efficient inoculum for the co-digestion of filter cake and bagasse, since very similar methane and biogas yields were obtained under the steady-state phase, independent of the inoculation strategy. The bacterial and methanogenic communities were also very similar at the end of the experiment, regardless of whether the reactors had been inoculated with FCM or MIX. Bacterial composition and succession showed that the major phyla involved in the anaerobic degradation of the waste products from the Brazilian bioethanol/sugar industry were *Bacteroidetes*, *Firmicutes* and *Synergistetes*. The co-digestion of filter cake and bagasse in both inocula setups led to the development of polysaccharide-degrading specialists affiliated to the genera *Prevotella* and *Bacteroides* (both comprised around 50 % relative abundance of the bacterial community at steady phase). Methanogenic communities varied mainly in the first 3 weeks of operation for co-digestion reactors and in the first 5 weeks for mono-digestion. The key methanogens were affiliated with *Methanosarcina*, *Methanobacterium*, *Methanoregulaceae* and *Methanomassiliicoccus* in co-digestion reactors, as opposed to *Methanosarcina*, *Methanobacterium*, *Methanoregulaceae* and a non-identified taxon in mono-digestion reactors. The most important reactor parameter correlating with the methanogenic community structure in both digestion setups was the pH. Stable isotope fingerprinting showed clearly, especially in case of carbon dioxide, the influence of different inoculum strategies and substrate feeding on the methanogenic activity. Based on the isotope analysis, in agreement with the molecular approach, both aceticlastic and hydrogenotrophic methanogenesis pathways contributed importantly to methane production throughout the experiment.

## Additional files

10.1186/s13068-016-0548-4Duplicate T-RFLP profiles of the methanogenic community dynamics for each reactor in order to show the reproducibility of the T-RFLP approach. **Figure S2.** Rarefaction curves of the pyrosequencing data of the 16S ribosomal RNA genes from the four co-digestion reactors (R3.5, R3.6, R3.7 and R3.8) at three different sampling points along the experiment. **Table S1.** Beta diversity index showing the community similarities between samples. **Figure S3.** 3D PCA diagram of the beta diversity. **Figure S4.** N-MDS plot showing the Bray–Curtis similarity of the methanogenic communities in parallel reactors.
10.1186/s13068-016-0548-4 List of obtained OTUs for the co-digestion reactors at three different sampling points.

## Abbreviations

AD: anaerobic digestion
FCM: fresh cattle manure
GC: gas chromatograph
HRT: hydraulic retention time
NH_4_–N: ammonium–nitrogen
NMDS: non-metric multidimensional scaling
MIX: engineered mixed inoculum
OLR: organic loading rate
OTUs: operational taxonomic units
T-RFLP: terminal restriction fragment length polymorphism
VOA: volatile organic acid
VOA/TIC: volatile organic acids per total inorganic carbonate buffer
