# Supplementary material for: Lessons learned from the microbial ecology resulting from different inoculation strategies for biogas production from waste products of the bioethanol/sugar industry
Source: Biotechnol Biofuels. 2016 Jul 16;9:144. doi: 10.1186/s13068-016-0548-4 (PMC4947286; doi:10.1186/s13068-016-0548-4)
Supplement: Supplementary file 1 — 10.1186/s13068-016-0548-4Duplicate T-RFLP profiles of the methanogenic community dynamics for each reactor in order to show the reproducibility of the T-RFLP approach. Figure S2. Rarefaction curves of the pyrosequencing data of the 16S ribosomal RNA genes from the four co-digestion reactors (R3.5, R3.6, R3.7 and R3.8) at three different sampling points along the experiment. Table S1. Beta diversity index showing the community similarities between samples. Figure S3. 3D PCA diagram of the beta diversity. Figure S4. N-MDS plot showing the Bray–Curtis similarity of the methanogenic communities in parallel reactors. [file 13068_2016_548_MOESM1_ESM.docx]

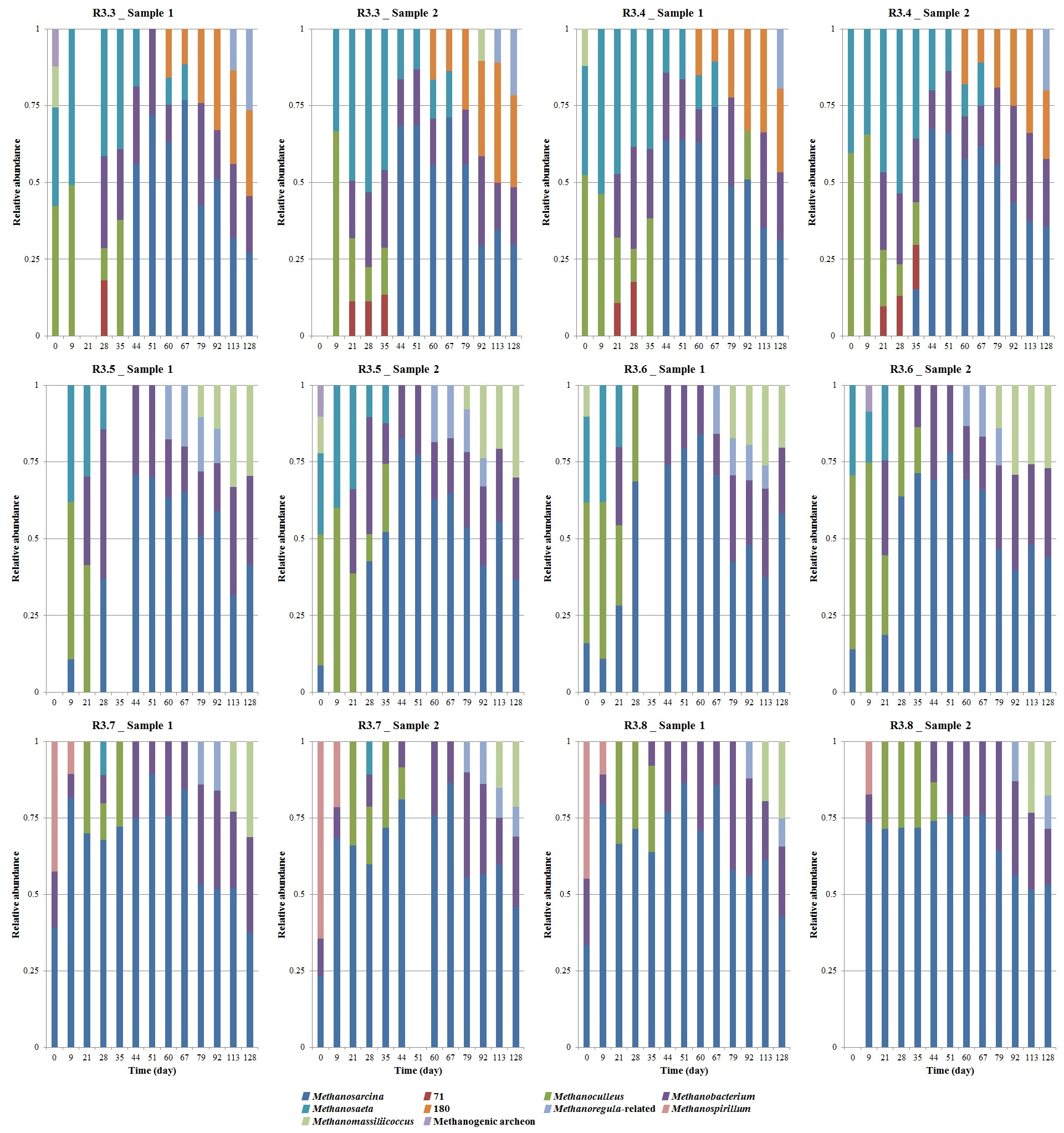


Supplementary Figure S1. Duplicate T-RFLP profiles of the methanogenic community dynamics for each reactor in order to show the reproducibility of the T-RFLP approach.


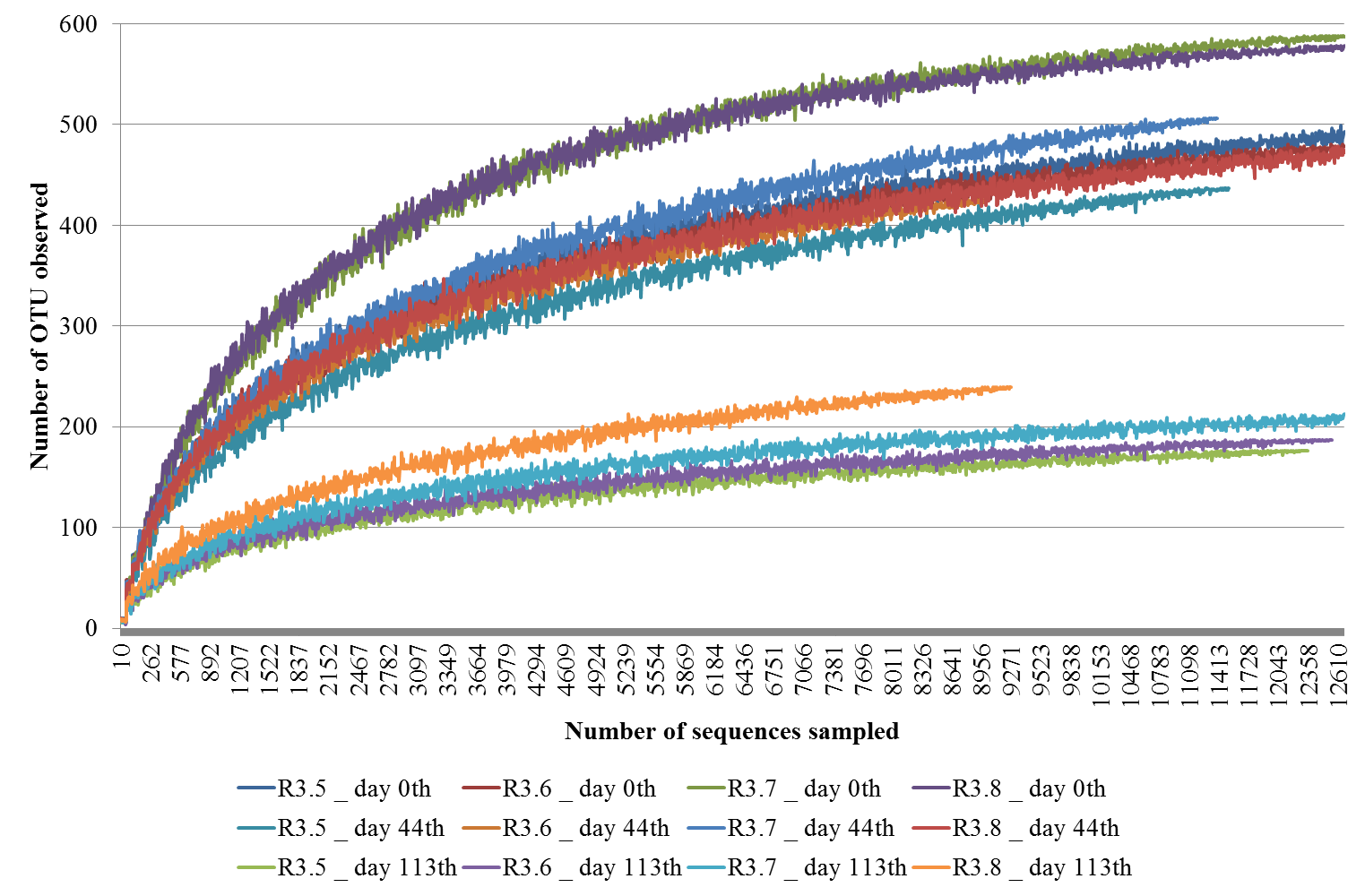


Supplementary Figure S2. Rarefaction curves of the pyrosequencing data of the 16S ribosomal RNA genes from the four co-digestion reactors (R3.5, R3.6, R3.7 and R3.8) at three different sampling points along the experiment.

Supplementary Table S1. Beta diversity indices based on 16S rRNA gene amplicon sequences showing the community similarities between samples.

|  | | Day 0 | | | | Day 44 | | | | Day 113 | | | |
| --- | --- | --- | --- | --- | --- | --- | --- | --- | --- | --- | --- | --- | --- |
|  |  | R3.5 | R3.6 | R3.7 | R3.8 | R3.5 | R3.6 | R3.7 | R3.8 | R3.5 | R3.6 | R3.7 | R3.8 |
| Day 0 | R3.5 | -0.0087 | 0.0265 | 0.0110 | -0.0067 | -0.2098 | 0.1676 | 0.1964 | 0.1496 | -0.0073 | -0.0659 | 0.0386 | -2.70E-09 |
|  | R3.6 | -0.0121 | 0.0147 | 0.0080 | -0.0033 | -0.2560 | 0.1325 | 0.0481 | 0.2478 | 0.0082 | 0.0607 | -0.0391 | -2.70E-09 |
|  | R3.7 | 0.0143 | -0.0193 | -0.0118 | -0.0246 | -0.2830 | -0.3782 | 0.0503 | -0.0446 | -0.0227 | 0.0491 | 0.0503 | -2.70E-09 |
|  | R3.8 | 0.0083 | -0.0041 | 0.0041 | 0.0367 | -0.2858 | -0.3787 | 0.0403 | -0.0580 | 0.0181 | -0.0460 | -0.0510 | -2.70E-09 |
| Day 44 | R3.5 | 0.0963 | -0.1116 | 0.0610 | -0.0733 | -0.0533 | 0.2056 | 0.0437 | -0.1497 | 0.0293 | 0.0014 | -0.0108 | -2.70E-09 |
|  | R3.6 | 0.0500 | -0.0626 | -0.0760 | 0.0951 | -0.0440 | 0.2502 | 0.0461 | -0.1116 | -0.0385 | 0.0125 | 0.0008 | -2.70E-09 |
|  | R3.7 | -0.0964 | 0.0405 | 0.1058 | 0.0492 | -0.1455 | 0.1062 | -0.2145 | -0.0675 | -0.0193 | 0.0024 | 0.0135 | -2.70E-09 |
|  | R3.8 | -0.0552 | 0.0630 | -0.0974 | -0.0642 | -0.2067 | 0.1219 | -0.2087 | -0.0591 | 0.0260 | -0.0147 | -0.0012 | -2.70E-09 |
| Day 113 | R3.5 | -0.1344 | 0.0070 | -0.0065 | 0.0237 | 0.4046 | -0.0215 | 0.1460 | -0.0582 | 0.0864 | 0.0176 | 0.0093 | -2.70E-09 |
|  | R3.6 | -0.0786 | -0.1341 | -0.0132 | -0.0232 | 0.3983 | -0.1198 | -0.1162 | 0.1384 | -0.0492 | -0.0195 | -0.0049 | -2.70E-09 |
|  | R3.7 | 0.0213 | 0.1308 | 0.0102 | -0.0381 | 0.3669 | -0.0039 | 0.1201 | -0.0932 | -0.0730 | 0.0064 | -0.0216 | -2.70E-09 |
|  | R3.8 | 0.1950 | 0.0493 | 0.0048 | 0.0288 | 0.3142 | -0.0818 | -0.1515 | 0.1060 | 0.0420 | -0.0041 | 0.0161 | -2.70E-09 |
|  | |  |  |  |  |  |  |  |  |  |  |  |  |
| eigvals | | 0.0873 | 0.0609 | 0.0309 | 0.0263 | 0.8956 | 0.4846 | 0.2108 | 0.1745 | 0.0213 | 0.0137 | 0.0093 | 8.73E-17 |
| % variation explained | | 4.3334 | 3.0200 | 1.5319 | 1.3069 | 44.4419 | 24.0463 | 10.4624 | 8.6594 | 1.0586 | 0.6785 | 0.4606 | 4.33E-15 |

Legend: The values were obtained using unweighted UniFrac.


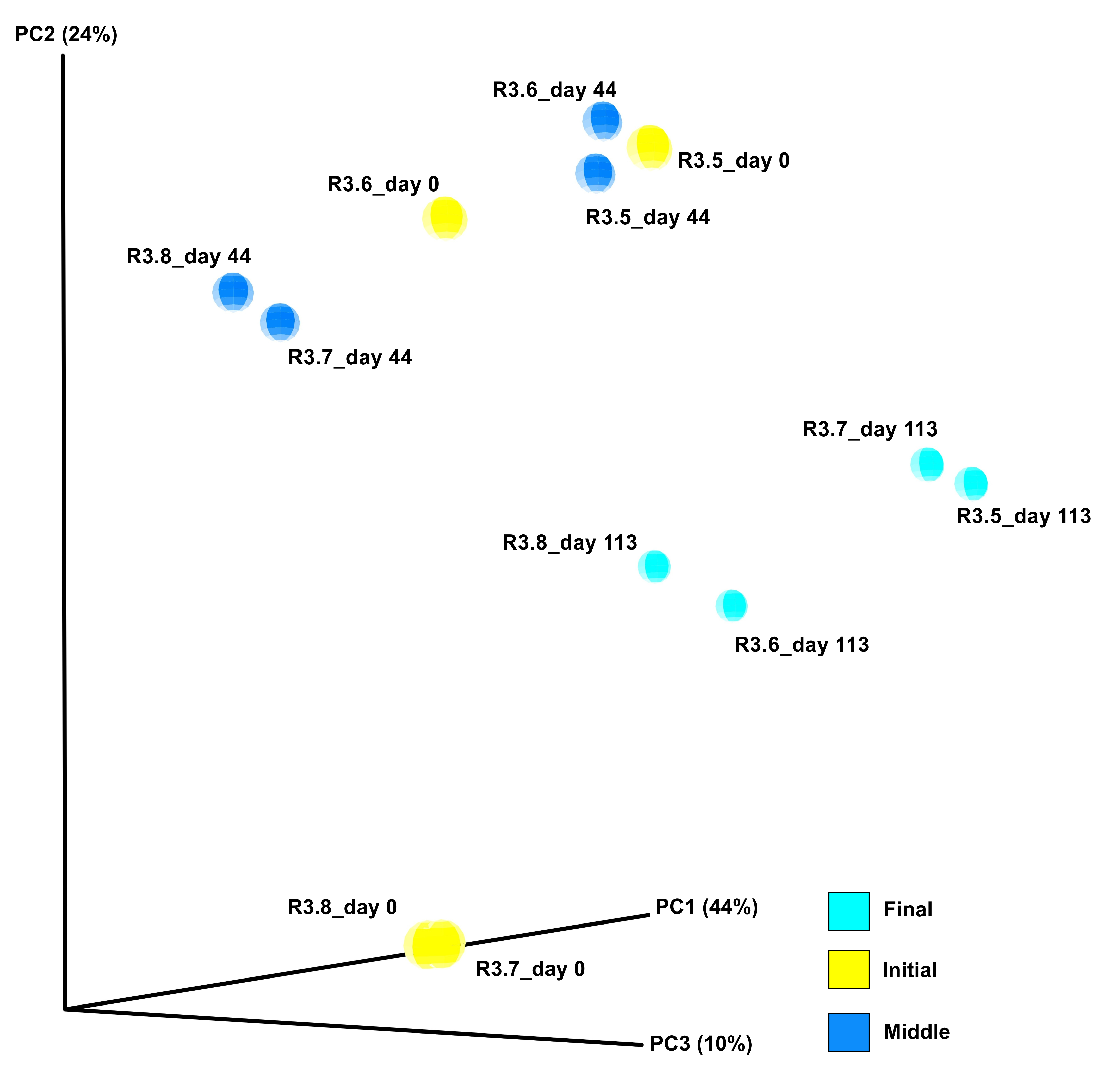


Supplementary Figure S3. Two dimensional version of 3D principal component analysis diagram based on the beta diversity of bacterial communities in various reactor samples taken at different time points.


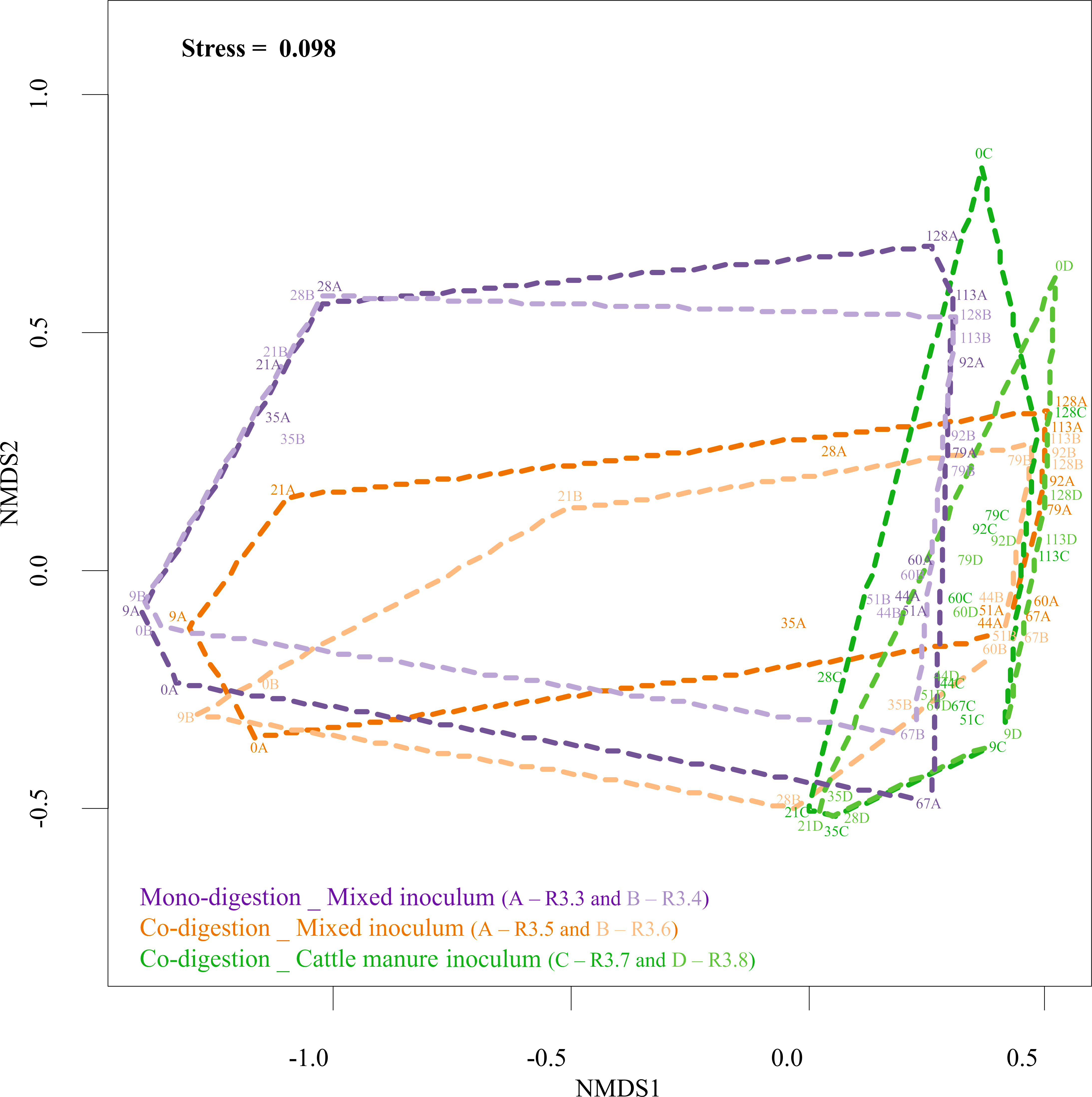


Supplementary Figure S4. N-MDS plot showing the similarity of the methanogenic community compositions in parallel reactors based on the Bray-Curtis dissimilarity index.
